# Supplementary material for: PKM2 Modulation in Head and Neck Squamous Cell Carcinoma
Source: Int J Mol Sci. 2022 Jan 11;23(2):775. doi: 10.3390/ijms23020775 (PMC8775697; doi:10.3390/ijms23020775)
Supplement: Supplementary file 1 [file ijms-23-00775-s001.zip › ijms-1508895 - Supplementary.pdf]

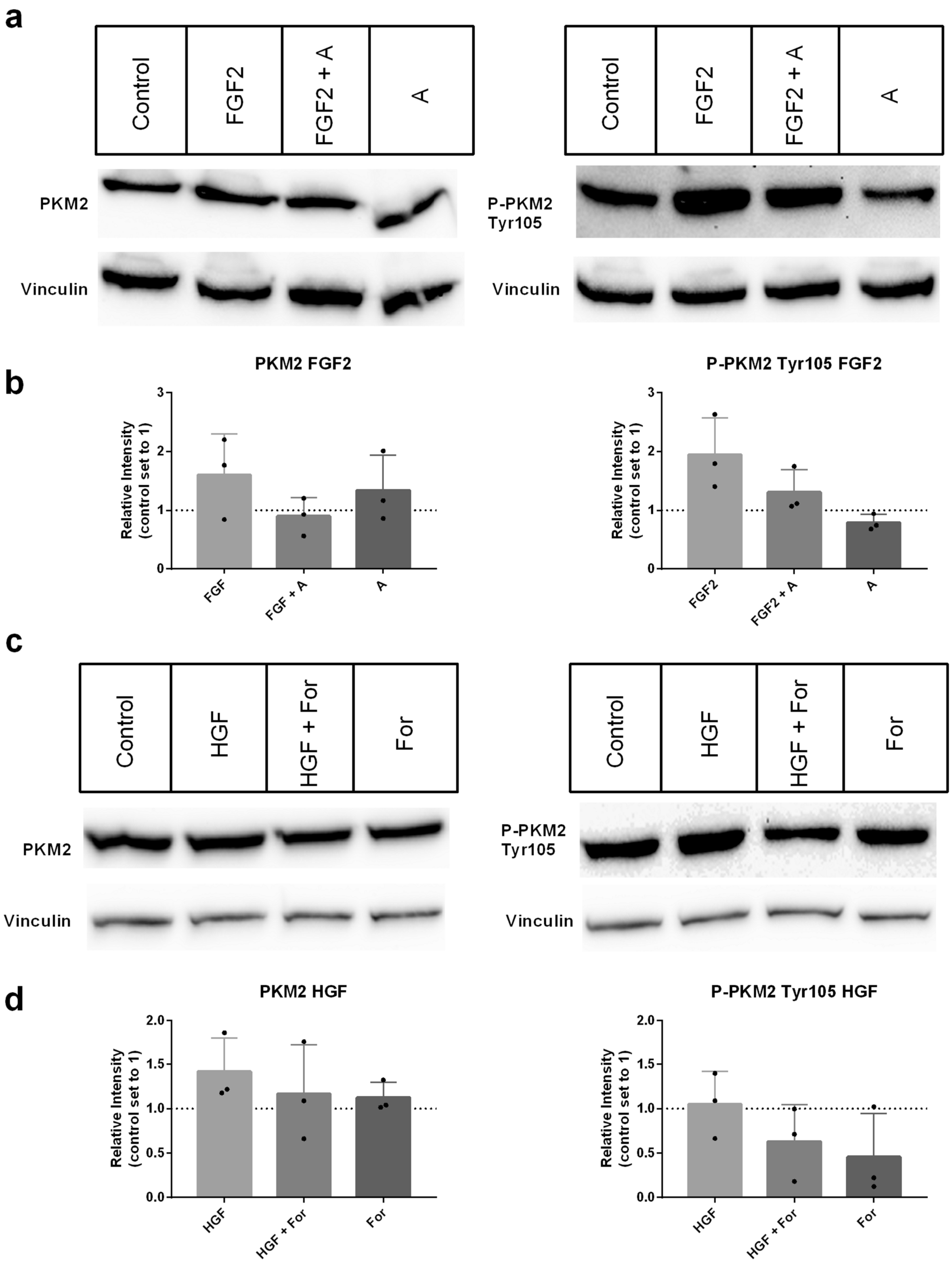

Figure S1: Influence of growth factors FGF2 and HGF on PKM2. (a) PKM2(left) or P-PKM2 Tyr105 (right) specific Western-Blots of Detroit 562 cell-lysates stimulated for 48 hours with 50 nM FGF2, 50 nM FGF2 in presence of 10 µM AZD4547 (FGF2 + A), AZD4547 only (A) or with vehicle alone (Control). Vinculin was used as a loading control. (b) Western-Blot intensities relative to untreated control. Shown are mean values and SD of three independent experiments as shown in (a). Vinculin

intensities were used for normalization. (c) As shown in (a) but cells stimulated with 0.7 nM HGF, 0.7 nM HGF in presence of 0.5 µM Foretinib (HGF + For), Foretinib alone (For) or with vehicle alone (Control). (d) Western-Blot intensities relative to untreated control. Shown are mean values and *SD* of three independent experiments as shown in (c).

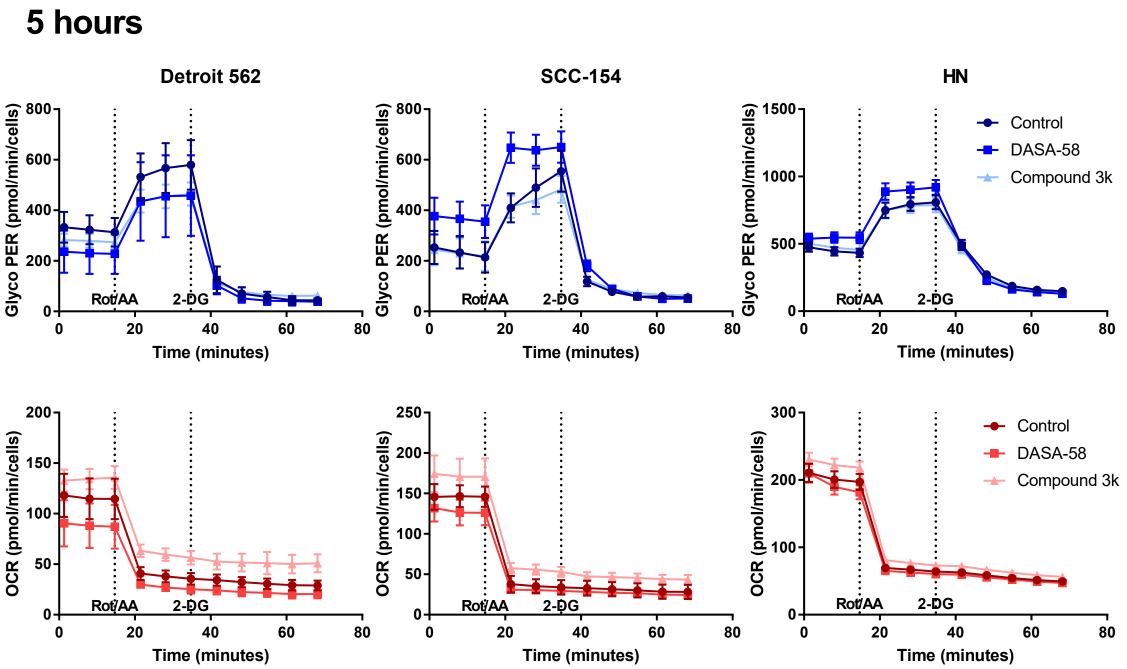

Figure S2: Results of glycolytic rate assays performed with different HNSCC cell lines after 5 hours of stimulation with Compound 3k, DASA-58 or with vehicle alone (Control). GlycoPER kinetic graphs in blue, corresponding OCR kinetic graphs below in red. Dotted lines indicate time points of injections of Rotenone in combination with Antimycin A (Rot/AA) and 2-Desoxy-D-Glucose (2-DG).

0.5 hours

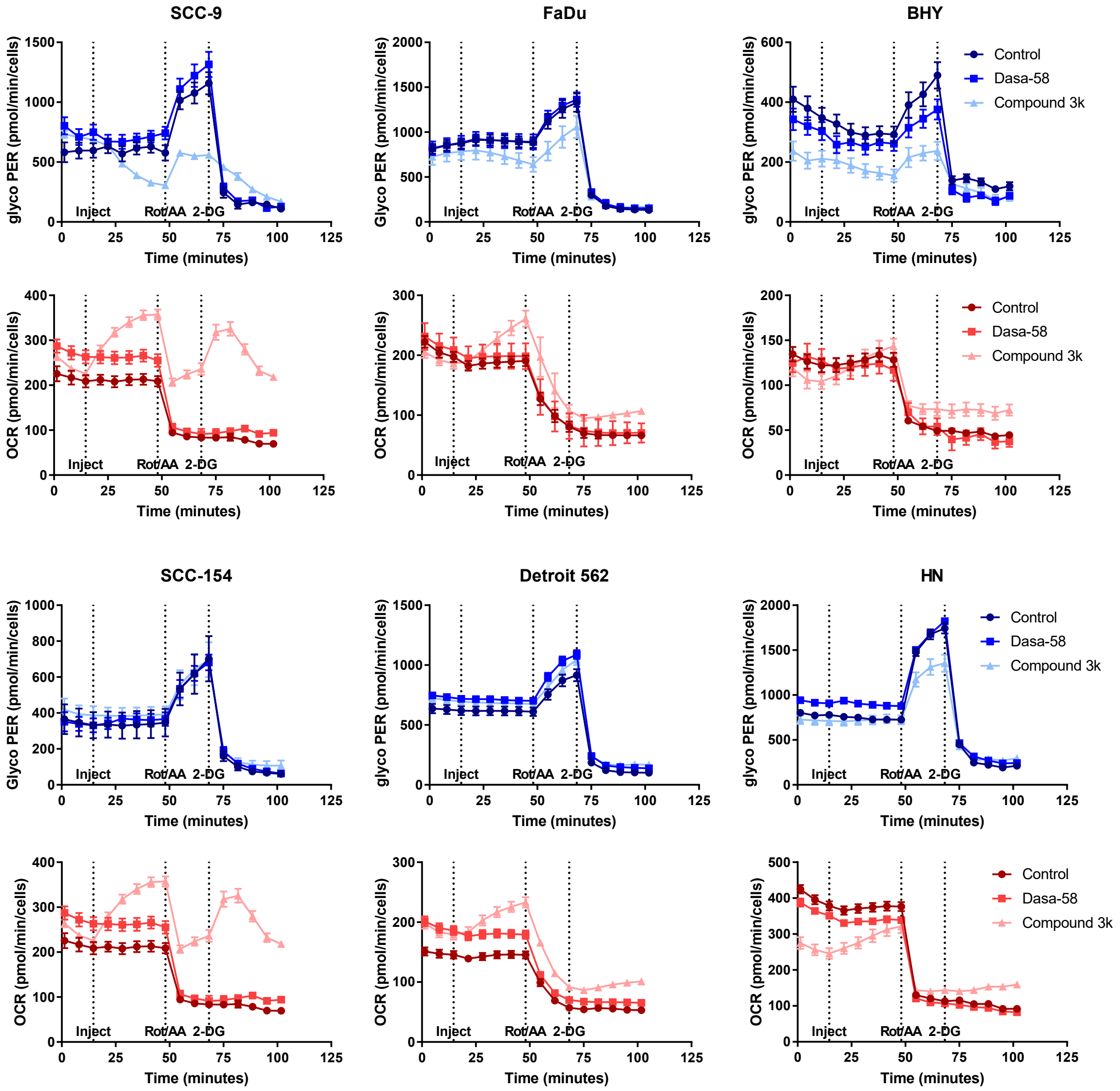

Figure S3: Glycolytic rate assays after 0.5 hours of DASA-58 and Compound 3k incubation. Different HNSCC cell lines were pre-injected (Inject, dotted line on the left) with 30 µM Compound 3k, 30 µM DASA-58 or with vehicle alone (Control). Rotenone/Antimycin A (Rot/AA) injection (dotted line in the middle) followed after 5 additional time points of measurement, resulting in an incubation time of Compound 3k and DASA-58 of 30 minutes. Dotted line on the right indicates time point of injection of 2-Desoxy-D-Glucose (2-DG). Glyco PER kinetic graphs in blue, corresponding OCR kinetic graphs below in red.

16 hours

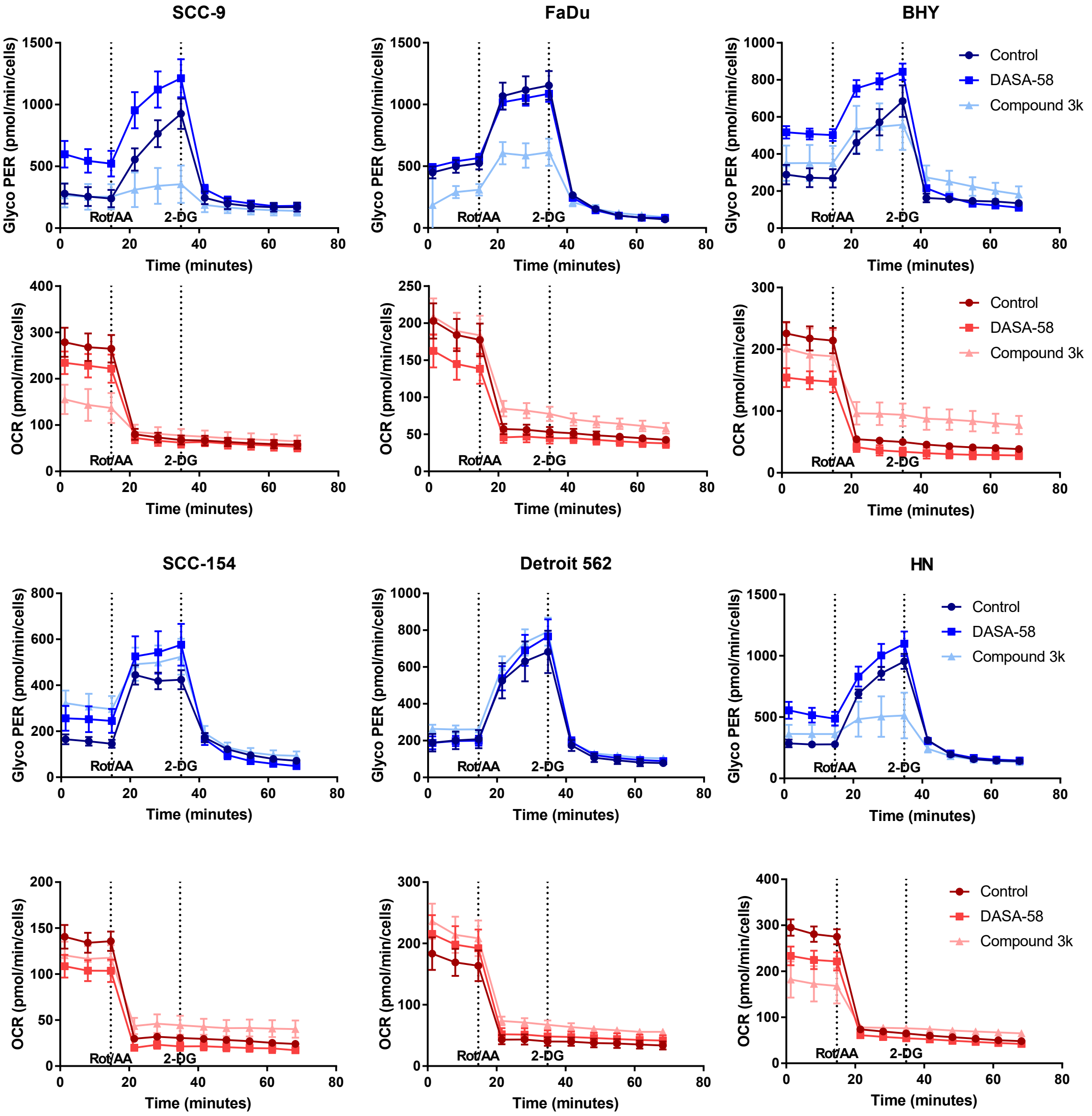

Figure S4: Results of glycolytic rate assays after 16 hours of incubation with DASA-58 and Compound 3k. Different HNSCC cell lines were incubated for 16 hours with 30 µM Compound 3k, 30 µM DASA-58 or with vehicle alone (Control). Glyco PER kinetic graphs in blue, corresponding OCR kinetic graphs below in red. Dotted lines indicate time points of injections of Rotenone in combination with Antimycin A (Rot/AA) and 2-Desoxy-D-Glucose (2-DG).

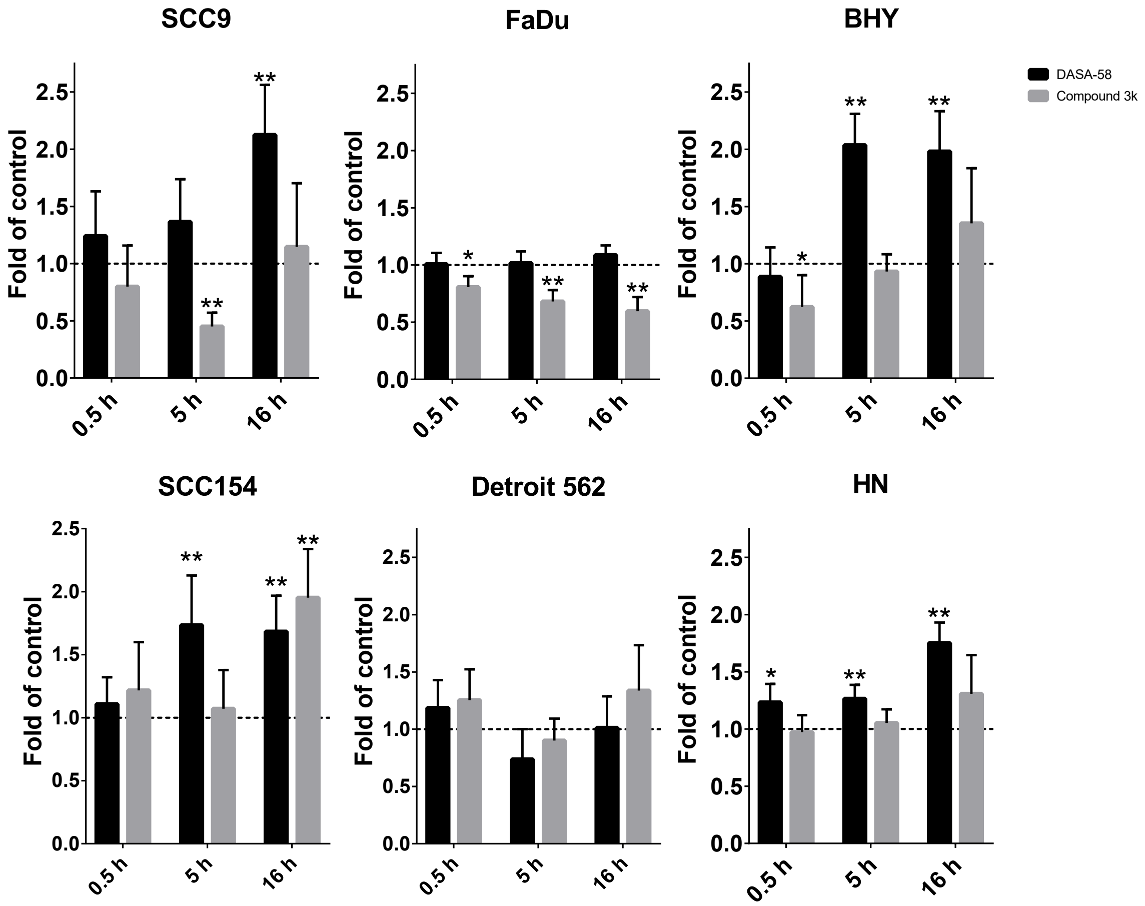

Figure S5: Overview of basic glycolysis of HNSCC cell lines after different duration of DASA-58 and Compound 3k treatment. Basic glycolysis (corresponds to the Glyco PER value of data point 3 in the kinetic graphs shown in figure 4b, S2 and S4 and of data point 8 in figure S3) after 0.5, 5h and 16 hours of treatment with 30 µM Compound 3k or DASA-58 of all six investigated HNSCC cell lines. Shown are changes in relation to the vehicle treated control. Data points represent means with *SD*, *n* = 10. *: p<0.01, **: p<0.001, one sample t-test.
